# Framework for virtual education of COVID-19 vaccines for Mandarin-speaking learners: an educational intervention module

**DOI:** 10.12688/mep.19207.1

**Published:** 2023-03-08

**Authors:** JiCi Wang, Benjamin M. Moy, Ross T. Kaufhold, Aurelio Muzaurieta, Yang Xia, Shannon Jiang, Angela Yim, Jane Chang Miller, Shiwei Zhou, Pearl Lee, Lisa Hou, Janilla Lee, Michael Heung

**Affiliations:** 1 University of Michigan Medical School, 1301 E. Catherine Street, Ann Arbor, MI, 48109, USA; 2 Michigan Medicine 3914, Taubman Center, 1500 E. Medical Center Drive, Ann Arbor, MI, 48109, USA; 3 Asian Center-Southeast Michigan, Southfield, MI, 48075, USA

**Keywords:** medical, education, COVID-19, vaccines, Mandarin, Chinese, virtual, module

## Abstract

Background: In the United States, patients with limited English proficiency face significant barriers to comprehending and acting upon health-related information, particularly during the COVID-19 pandemic. The ability of health professionals to communicate COVID-19-related information to Mandarin-speaking patients has proved critical in discussions about vaccine efficacy, side effects, and post-vaccine protection.

Methods: The authors created a one-hour educational module to help Mandarin-speaking medical students better convey COVID-19 vaccine information to Mandarin-only speakers. The module is composed of an educational guide, which introduced key terminology and addressed commonly asked questions, and pre- and post-surveys. The authors recruited 59 Mandarin-speaking medical students all of whom had previously completed a medical Mandarin elective. The module and surveys were distributed and completed in August 2021. Data analysis measured the change in aggregate mean for subjective five-point Likert-scale questions and change in percent accuracy for objective knowledge-based questions.

Results: The educational module significantly improved participants' subjective comfort level in discussing the COVID-19 vaccine in English and Mandarin. The largest improvement in both English and Mandarin was demonstrated in the participants ability to explain differences between the COVID-19 vaccines, with an aggregate mean improvement of 0.39 for English and 1.48 for Mandarin. Survey respondents also demonstrated increased percent accuracy in knowledge-based objective questions in Mandarin.

Conclusions: This module provides Mandarin-learning medical students with skills to deliver reliable information to the general population and acts as a model for the continued development of educational modules for multilingual medical professionals.

## Introduction

Limited English proficiency is known to be a major barrier to accessing quality healthcare within the United States ^1^. Language barriers have contributed to a lack of access to accurate and updated healthcare information, a disparity that has been exacerbated by the COVID-19 pandemic ^2^. Chinese-speaking communities comprise a large portion of the United States population, over 3.3 million U.S. residents, of which 55% meet the criteria for limited English proficiency ^3^. This disparity in health literacy has created uncertainty and confusion around topics related to SARS-CoV-2/COVID-19, especially vaccination efficacy, side effects, and post-vaccine protection. Cooperation between medical professionals and community groups has proved essential throughout the pandemic, particularly in delivering critical information to individuals in their native or preferred language ^4, 5^.

In March 2021, Asian Center-Southeast Michigan, a non-profit organization serving the Asian-American/Pacific Islander community in metropolitan Detroit, contacted our group of Mandarin-speaking medical students at the University of Michigan Medical School to request a presentation in Mandarin about the COVID-19 vaccines for their Mandarin-speaking community members. While preparing the presentation, we realized that a niche set of medical terminology not covered in traditional medical language electives would be required to comprehensively communicate vaccine information. Additionally, few educational resources were available in Mandarin to assist learners to acquire the requisite vocabulary for conveying information about the COVID-19 vaccines. However, since all the presenters had previously completed the Medicine in Mandarin elective, we possessed adequate background skills to learn new medical terms necessary for effectively communicating information about the COVID-19 vaccines to Mandarin-only speakers ^6^. We worked with the Medicine in Mandarin elective instructor at the University of Michigan as well as Mandarin-speaking staff at Asian Center-Southeast Michigan to identify, learn, and practice relevant terminology. We incorporated this set of vocabulary with previously learned content to accurately convey healthcare information during the presentation to community members in April 2021.

After the presentation, we created an educational module to share our group’s experiences with other Mandarin-speaking medical students to improve communication of COVID-19 vaccine information to Mandarin-only speakers. Previous research has shown that Chinese language workshops and educational materials like phrasebooks allow medical students to refine their linguistic abilities and help to increase students’ perceived confidence in communicating with Mandarin-speaking patients ^7, 8^. Additionally, peer-led and peer-developed curricula can readily identify knowledge gaps in an educational cohort while providing a collaborative space for language learning ^7^. Our module included a table of key terms, responses to ten relevant “commonly asked questions,” and pre- and post-surveys to evaluate the effect of our intervention.

This study seeks to evaluate the efficacy of an educational module designed to assist Mandarin-speaking medical students with acquiring and utilizing Mandarin terminology regarding COVID-19 vaccine-related information. Our educational guide acts as a model for developing topical phrasebooks or modules that can assist multilingual medical students with learning and practicing important terminology that they will use during conversations with patients. We believe that our instructional module addresses a gap in the medical language discipline by providing a guide for professional students to better tackle the language barrier that limits access to quality information amid the uncertainty of the COVID-19 pandemic.

## Methods

### Study design

The study was designed as an educational intervention via an online module, with participants’ comfort level with discussing vaccine-related information being analyzed before and after engagement with the module. The location of research was centered in Ann Arbor, Michigan, though participants engaged remotely from all across the United States. The educational module was designed to be completely virtual. Surveys and corresponding data were collected for a two-week period in August 2021. Data was processed and analyzed by the research team in Ann Arbor, Michigan.

### Study population

To normalize medical Mandarin knowledge and minimize selection bias, we limited our study population to students who had previously enrolled in and completed a medical Mandarin course at their respective medical schools. This requirement operated as a baseline for ensuring at least intermediate to advanced proficiency in Mandarin Chinese. Medical Mandarin electives are offered at medical schools across the nation and aim to teach accurate Mandarin terminology for use during patient encounters and introduce cultural values so that students provide culturally competent care for Mandarin-speaking patients.

Survey respondents were identified by consulting student rosters and email distribution networks for medical Mandarin electives at medical schools across the country. Our module was distributed via email to medical students at seven U.S. academic medical centers. Participants were identified by personal connections with Mandarin-speaking medical students at other academic medical centers. Participants were encouraged to distribute the module to other medical students who met the study’s inclusion criteria. Inclusion criteria were limited to students who had completed an elective in medical Mandarin at a medical school. A study size of at least thirty participants was determined to ensure adequate student representation and data significance. Participants were given two weeks to complete the module. The module was designed to be completed within one hour. The first fifty survey respondents were each compensated $40 for completion of the pre-survey, educational guide, and post-survey. Funding was provided through the University of Michigan Medical School M1 Summer Impact Accelerator Grant. Compensation could have been a source of potential bias in this study.

### Ethical statement

The study was exempt from regulation by the University of Michigan Institutional Review Board (HUM00200622), as it involved a benign intervention limited to responses by participants via online surveys. Participants’ names and email addresses were collected for the distribution of compensation and were otherwise de-identified for all research purposes. All participants were required to sign a consent form agreeing to collection of information including name, email address, and personal demographics. Participants were informed that their information would be de-identified as part of data processing. All participants provided consent prior to enrollment by electronically signing at the beginning of both pre- and post-surveys.

### Data collection

In engaging with our module, students were instructed to complete a survey before and after reading through a bilingual English-Mandarin educational guide that contained learning objectives, a table of key vaccine terms, and information regarding 10 commonly asked questions that we heard from the local community. A copy of the survey can be found under *Extended data* ^9^. Our guide synthesized information from government sources, peer-reviewed research, and Michigan Medicine faculty. Our targeted educational module evaluated participants’ knowledge and fluency of vaccine-related information in Mandarin via scores on the two surveys. The pre-guide and post-guide surveys were drafted on Qualtrics. The pre-survey contained questions about participants’ demographics and language backgrounds. Both surveys included six questions regarding participants’ comfort levels using English to discuss each of the learning objectives identified in the educational guide, and eight questions regarding participants’ comfort levels using Mandarin to discuss each of the learning objectives. The questions were identical in pre- and post-surveys to measure a change in participants’ comfort levels in using both English and Mandarin for each learning objective before and after intervention (Appendix B). Three optional free-text questions were provided in the post-survey for participants to leave feedback about the educational module. The module was reviewed by Mandarin-speaking medical faculty, a Mandarin healthcare interpreter, and two native Mandarin speakers from Asian Center-Southeast Michigan to ensure accuracy of the medical content and translation.

### Analysis

We analyzed data for the Likert-scale questions using R Studio and created stacked bar plots for pre- and post-survey responses for each learning objective, categorized by English vs Mandarin. Responses were recoded into numerical values: strongly disagree = 0, somewhat disagree = 1, neither agree nor disagree = 2, somewhat agree = 3, strongly agree = 4 to allow measurement of change in mean. Scores before and after the intervention were compared using paired t-tests. The final analysis represented an aggregate change before and after intervention for each question regarding English and Mandarin comfort levels addressing each learning objective.

Data analysis for knowledge-based questions was completed using the GraphPad Prism 9 for visual representation and Chi-squared analysis. Bar plots were created for pre- and post-survey responses to four objective content multiple-choice questions. Two of the questions (6 and 7) allowed for multiple answer selection and included more than one correct answer. For these questions, each answer option was evaluated pre- and post-survey via Chi-squared analysis to assess for significant changes in answer selection after our intervention. For questions that had a single correct answer (Questions 5 and 8), only the participant selection of the correct answer was included in our data analysis.

## Results

Fifty-nine students across the United States participated in the study, all of whom had completed a medical Mandarin elective. Participants represented varying classes in medical school, and over 86% of students reported English as their first language. Participants learned Mandarin from a variety of sources, with the most frequent being parents/family life, followed by weekend Mandarin classes, secondary or post-secondary education, and self-study. The full dataset can be found under *Underlying data* ^9^.

Participants’ goals for learning Mandarin included improving communication with Mandarin-speaking patients and family members, learning about personal heritage/culture, and pursuing an international career in a Mandarin-speaking country. Nearly half of the participants expressed interest in working with a community and/or patient population that is mainly Mandarin-speaking ( Table 1).

**Table 1.** Student demographic information

**Participant Demographic Information**	
Total Number of Students	59
**Year in Medical School**	
M1	5.1%
M2	30.5%
M3	25.4%
M4	18.6%
MD/PhD	11.9%
Graduated	3.5%
Not yet enrolled	5.1%
**Primary Language**	
English	86.4%
Fully Bilingual (English and Mandarin)	10.2%
Other	1.7%
**Origin of Mandarin Knowledge**	
Parents/Home	76.3%
Born in a Mandarin-speaking country and attended school there	11.9%
School (High school, college, medical school)	45.7%
Weekend Mandarin School	50.8%
Study abroad	8.5%
Self-study	16.9%
Other	5.1%

**Figure 1.**
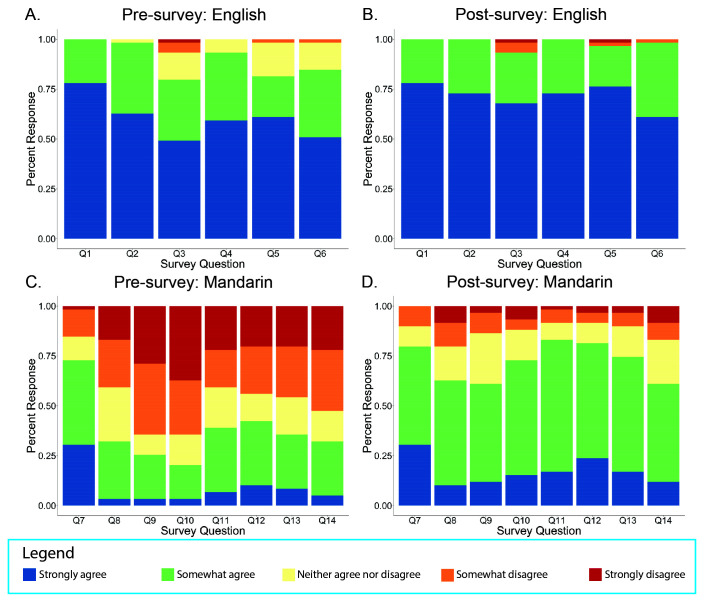
Response distribution to pre-and post-survey questions addressing participants’ comfort levels for discussing learning objectives in English and Mandarin from our virtual educational module for Mandarin-speaking medical students.

This educational module significantly improved participants’ subjective comfort level in discussing the COVID-19 vaccine in both English and Mandarin. Participants’ pre- and post-survey subjective responses to various questions were recorded on a Likert-scale from “strongly agree” to “strongly disagree” ( Figure 1). Comparing the pre- and post-survey responses, there was a statistically significant change in the mean response towards “strongly agree” on Questions 2 through 6 for discussing questions in English and Questions 2 through 8 for discussing questions in Mandarin ( Figure 1).

Specifically, after completing the module, students felt more comfortable using English to explain how COVID-19 vaccines protect against COVID-19 (Q2 English), the differences between available COVID-19 vaccines (Q3 English), side effects of the COVID-19 vaccines (Q4 English), the timeframe for COVID-19 vaccines to be effective (Q5 English), and what activities are recommended by the CDC after receiving the vaccine (Q6 English). Regarding understanding and explaining concepts in English, students demonstrated a 0.12 aggregate mean increase in comfort levels for Q2 (p<0.05), 0.39 increase for Q3 (p<0.01), 0.21 increase for Q4 (p<0.01), 0.30 increase for Q5 (p<0.01), and 0.25 increase for Q6 (p<0.01). The largest difference in mean score was observed for participants’ comfort levels with understanding the differences between the available COVID-19 vaccines in English (Q3), in which the aggregate mean of 3.20 increased to 3.59 post-intervention ( Table 2).

**Table 2.** Change in aggregate mean for each learning objective in English and Mandarin. The table demonstrates the change in aggregate mean for each learning objective in both English and Mandarin. Participant responses were recoded into numerical values 0-4 (strongly disagree-strongly agree) to calculate aggregate mean score, with a shift toward more positive values, indicating greater comfort level with learning objectives. Statistical significance calculated using paired t-test. * indicates P-value <0.05, ** <0.01, *** <0.001, **** any value beyond <0.0001. NS denotes not significant.

Question	Pre-Survey Response Mean	Post-Survey Response Mean	Change in Mean between Pre- and Post-Surveys	P-value
Participants’ comfort level with **English**				
**Q1**: I feel comfortable conversing with others (family, friends, patients) using medical terminology in ENGLISH while still using patient-friendly language.	3.78	3.78	0	NS
**Q2**: I feel comfortable using ENGLISH to explain how COVID-19 vaccines protect against COVID-19 using patient-friendly language.	3.61	3.73	0.12 *	<0.05
**Q3**: I feel comfortable using ENGLISH to explain the differences between available COVID-19 vaccines (eg. Pfizer vs J&J) to patients, friends, and family.	3.20	3.59	0.39 ***	<0.001
**Q4**: I feel comfortable using ENGLISH to explain the side effects of the COVID-19 vaccines.	3.52	3.73	0.21 ***	<0.001
**Q5**: I feel comfortable using MANDARIN to explain the side effects of the COVID-19 vaccines.	3.41	3.71	0.30 ***	<0.001
**Q6**: I feel comfortable using ENGLISH to discuss what activities are recommended by the CDC after receiving the COVID-19 vaccine.	3.34	3.59	0.25 **	<0.01
Participants’ comfort level with **Mandarin**				
**Q7**: I feel comfortable conversing with others (family, friends, patients) in conversational MANDARIN.	2.86	3.00	0.14	NS
**Q8**: I feel comfortable conversing with others (family, friends, patients) using medical terminology in MANDARIN while still using patient-friendly language.	1.78	2.44	0.66 ****	<0.0001
**Q9**: I feel comfortable using MANDARIN to explain how COVID-19 vaccines protect against COVID-19 using patient-friendly language.	1.36	2.56	1.2 ****	<0.0001
**Q10**: I feel comfortable using MANDARIN to explain the differences between available COVID-19 vaccines (eg. Pfizer vs J&J) to patients, friends, and family.	1.22	2.70	1.48 ****	<0.0001
**Q11**: I feel comfortable using MANDARIN to explain the side effects of the COVID-19 vaccines.	1.83	2.90	1.07 ****	<0.0001
**Q12**: I feel comfortable using MANDARIN to explain the timeframe for COVID-19 vaccines to be effective.	1.88	2.93	1.05 ****	<0.0001
**Q13**: I feel comfortable using MANDARIN to discuss what activities are recommended by the CDC after receiving the COVID-19 vaccine.	1.78	2.78	1.00 ****	<0.0001
**Q14**: I understand how the COVID-19 vaccine is perceived by MANDARIN-speaking communities.	1.63	2.48	0.85 ****	<0.0001

After completing the module, students felt more comfortable using Mandarin to explain medical terminology (Q8 Mandarin), how COVID-19 vaccines protect against COVID-19 (Q9 Mandarin), the differences between available COVID-19 vaccines (Q10 Mandarin), side effects of COVID-19 vaccines (Q11 Mandarin), the timeframe for COVID-19 vaccines to be effective (Q12 Mandarin), what activities are recommended by the CDC after receiving the vaccine (Q13 Mandarin), and understanding how the COVID-19 vaccine is perceived by Mandarin-speaking communities (Q14 Mandarin). Regarding comprehension and explanation of vaccine-related concepts written in Mandarin, students demonstrated a 0.66 aggregative mean increase in comfort levels for Q8 (p<0.01), 1.2 increase for Q9 (p<0.01), 1.48 increase for Q10 (p<0.01), 1.07 increase for Q11 (p<0.01), 1.05 increase for Q12 (p<0.01), and 1 increase for Q13 (p<0.01), and 0.85 increase for Q14 (p<0.01). Similar to the largest improvement in mean score on the English portion of the intervention, participants displayed the most significant improvement in mean score for using Mandarin to explain the differences between available COVID-19 vaccines (Q10) ( Table 2).

Compared to baseline, participants specifically reported an increased level of comfort for using both English and Mandarin to convey the vaccine-related information summated in our learning objectives. Overall, there was a significantly greater increase in mean scores for the Mandarin portion after participants engaged with the module with some mean score increases for the English portion as well.

There was no statistically significant difference between pre- and post-survey responses in English on Question 1, which measured students’ comfort conversing with others using medical terminology in English. There was also no statistically significant difference between the pre- and post-survey responses in Mandarin on Question 7, which assessed students’ comfort communicating with others in conversational Mandarin. These results were expected as the participants were English-speaking medical students who have enrolled in a medical Mandarin course during medical school. Given that this module focused on communicating COVID-19 vaccine information in Mandarin, it would not likely improve students’ comfort using medical terminology in English or students’ comfort in conversational Mandarin generally.

In addition to completing the Likert-scale questions, participants were asked four knowledge-based questions (Q15–Q18) in Mandarin under the section “Competency in communicating information regarding COVID-19 using Mandarin”. Q15 and Q18 had a single correct answer, and participants demonstrated a statistically significant improvement post-intervention for both questions (p<0.01). Q15 asked participants to select the set of Mandarin terms that were most relevant for describing how the COVID-19 vaccine protects against the virus. After engaging with the module, the percentage of participants who chose the correct answer of “immunity, antibody, injection, vaccine” increased by 20.3%. An increase of 25.4% was also observed for participants who selected the correct answer to Q18, which asked participants to select the best answer to the question translated to “Can you still spread the virus after getting vaccinated?”

**Figure 2.**
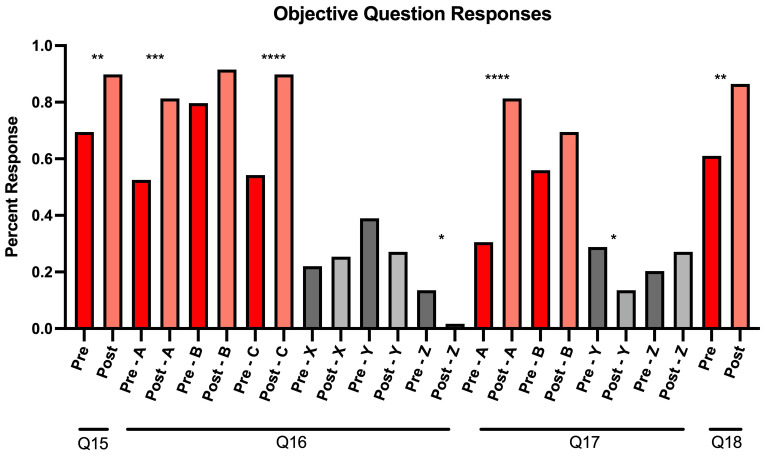
Response percentages for pre-and post-survey objective questions from our virtual educational module for Mandarin-speaking medical students. For Q16 and Q17, correct answers are graphically represented as answers A, B, and C, while incorrect answers were recorded as X, Y, and Z.

Students could select multiple correct answers for Q16 and Q17 ( Figure 2). Q16 asked participants to choose terms that described the side effects of the COVID-19 vaccine. Participants demonstrated a statistically significant improvement in selecting two of the correct answers, “fatigue” (p<0.001) and “low-grade fever” (p<0.0001) as well as not selecting one of the incorrect answers, “death” (p<0.05). Q17 asked participants to identify specific activities that the CDC deemed appropriate after receiving the vaccine as of the time of the survey in April 2021. There was a statistically significant increase in selecting one of the correct answers “Can travel internationally without quarantining after returning to America” (p<0.0001), and a significant improvement in not selecting one of the incorrect answers “Can attend medium or large gatherings” (p<0.05) ( Figure 2).

Overall, 97% of participants recommended this educational module to others who want to improve their abilities to communicate with and educate native Mandarin speakers about the COVID-19 vaccine in Mandarin. In addition, the optional free-response post-survey questions, Q19, Q20, and Q21 yielded a total of 107 unique responses that unveiled several themes ( Table 3). These included comments about the effectiveness of the module’s streamlined approach and incorporation of pinyin for pronunciation assistance as well as recommendations for incorporating audio recordings and mini quizzes for increased interaction.

**Table 3.** Response analysis of optional free-text questions from the post-survey highlighting several common themes.

**Q19: Which parts of the educational module helped to facilitate your learning? Which aspects of this module did you** **like?**
*Pinyin in the module was one of the most helpful things from the module* - “I liked the incorporation of pinyin into the module to help those of us who are fluent but have a more difficult time reading” - “Love the pinyin spelled out to block off certain words. Was very helpful to have it laid out to work through reading it for words I wasn't familiar with” *Usefulness of the FAQ format and the keywords table:* - “I think the FAQ style of the module was perfect” - “The key words table was very useful, since it allows me to string together my own sentences/ways to describe COVID19 vaccination”
**Q20: Which areas of the educational module can be improved? Which aspects of this module did you not like? Feel** **free to share any thoughts or suggestions!**
*Incorporating audio guide to help with pronunciation:* - “Embedding audio clips would also help in case people need pronunciation help” - “Would be helpful to have audio associated to hear the sentences” *Adding traditional Chinese characters would be helpful:* - “Perhaps add traditional characters as some communities use these more than simplified.” *More interaction within the module would have facilitated learning:* - “Could use an interactive component of the module to reinforce learning **”**
**Q21: Please use this space to share any thoughts/experiences you have about using Mandarin in your life and/or** **career!**
*Shared their experiences using Mandarin in their life and/or medical career and commented on the usefulness of the module:* - “I think this is helpful for talking with friends and family and for providers who are fluent and certified to provide care in Mandarin, but I won't be able to use this information in my practice as it would lead to worse care for my patients. I definitely need to use a certified interpreter when giving this information to patients” - “I used to work in a Chinatown clinic, and felt that the questions/responses are quite relevant and came up frequently in doctor-patient interactions.” - “Super helpful guide, I've come across these questions from my family and struggled to relay this information correctly in Chinese”

## Discussion

Through this study, we demonstrate that topical educational modules can improve medical students’ ease and comfort with communicating vaccine-related information to Mandarin-speaking patients. While many medical students were able to inform patients about COVID-19 vaccines in English, our module offered students appropriate instruction to convey that information to Mandarin speakers. Most participants reported significantly increased comfort with discussing vaccine-related information in Mandarin after completing the module. Additionally, participants reported greater comfort in explaining vaccine information in English, likely due to an increased fund of knowledge and vaccine updates provided by the Centers for Disease Control and Prevention and University of Michigan faculty. The largest increase in comfort levels for both English and Mandarin was for the learning objective addressing differences between the US-based COVID-19 vaccines (Pfizer, Moderna, Johnson & Johnson), indicating a potential gap in knowledge that this intervention helped to mitigate. The efficacy of the educational module was also demonstrated with increased accuracy in selecting correct answers to knowledge-based questions in Mandarin, indicating that participants’ knowledge base and ability to interpret health information in Mandarin had improved.

As a student-developed and student-led initiative, our team drew on personal experiences from the Medicine in Mandarin elective and basic science coursework at the University of Michigan Medical School to customize the educational module’s content, length, and scope to maximize accessibility to medical students. Participants noted the module’s point-by-point, self-paced structure, key terms table, and responses to 10 relevant “commonly asked questions” to be especially helpful. While many participants were already knowledgeable about the COVID-19 vaccines, we recognize that the true impact of our module centered around teaching students how to explain the information appropriately in Mandarin.

One limitation of our study is generalizability, as medical Mandarin electives may not be available to medical students across the nation, do not have a standardized curriculum, and cover a range of topics using various learning modalities. Although we required participants to have previously completed a medical Mandarin elective, individual participants still possess varying degrees of proficiency that may have impacted comprehension of the module’s content and subsequent performance on the survey. While our module improved participants’ comfort level and objective ability to communicate COVID-19 vaccine-related information in Mandarin, it is outside the scope of this work to measure the health outcomes of patients who engaged with module participants.

We encourage the continued production of similar educational resources regarding COVID-19 as the pandemic continues to evolve, as well as other medical topics of interest. These modules could incorporate extensive pedagogical approaches such as schemas for patient education, audio recordings, or mini-quizzes to check users’ comprehension as they progress through the module. Another possible area for research is the formulation of a standardized medical language curriculum that provides baseline levels of linguistic proficiency as agreed upon by health professionals and language experts. In the age of digital education, we should strive to improve access to education and relevant materials for multilingual students enrolled at institutions without official medical language electives. Although professional medical interpreters remain necessary, the ability for providers to communicate with non-English-speaking patients in their native language is beneficial not only for building rapport but also when interpreters are unavailable, particularly in low-resource settings. Finally, it is important to assess the clinical utility that multilingual communication has on patient encounters to more effectively determine how information-sharing occurs during these medical encounters. Improving language proficiency in multilingual students and providers will help improve health literacy in patients with limited English proficiency by ensuring adequate communication of health-related information and providing culturally appropriate care in an inclusive environment. The latter is crucial as patient populations in the United States grow increasingly diverse.

In the context of widespread vaccine hesitancy among racial and ethnic minority groups in the United States, it may also be prudent to assess whether receiving information about the COVID-19 vaccine in one’s preferred language increases the likelihood of taking the vaccine. Results of such a study might inform efforts to increase vaccination rates among patients with limited English proficiency.

Ultimately, this Mandarin educational module significantly improved participants’ subjective comfort level in discussing the COVID-19 vaccine with Mandarin-speaking patients. Our module provides Mandarin-learning medical students with the skills to convey crucial health-related content to patients and functions as a model for developing additional educational resources for multilingual students.

## Data Availability

Dryad: Framework for Virtual Education of COVID-19 Vaccines in Mandarin-Speaking Learners. https://doi.org/10.5061/dryad.7sqv9s4vr ^9^. This project contains the following underlying data: deID_PRE_Mandarin_COVID_Vaccine_Pre-survey.xlsx deID_POST_Mandarin_COVID_Vaccine_Post-survey.xlsx This project contains the following extended data: Survey Educational guide Mandarin survey translation Data are available under the terms of the Creative Commons Zero “No rights reserved” data waiver (CC0 1.0 Public domain dedication).
