# Supplementary material for: Framework for virtual education of COVID-19 vaccines for Mandarin-speaking learners: an educational intervention module
Source: MedEdPublish (2016). 2023 Aug 3;13:14. Originally published 2023 Mar 8. [Version 3] doi: 10.12688/mep.19207.3 (PMC10509786; doi:10.12688/mep.19207.3)
Supplement: Peer review history

# Reviewer report: Reviewer response for version 3

**DOI:** 10.21956/mep.21122.r34414

**Published:** 2023-08-17

**Authors:** Anna Chur-Hansen

**Affiliations:** 1 School of Psychology, Faculty of Health and Medical Sciences, University of Adelaide, Adelaide, SA, Australia

Now that the qualitative data have been removed, I am now happy to approve the manuscript.

Is the rationale for developing the new method (or application) clearly explained?

Partly

Is the description of the method technically sound?

Partly

Are the conclusions about the method and its performance adequately supported by the findings presented in the article?

Partly

If any results are presented, are all the source data underlying the results available to ensure full reproducibility?

Partly

Are sufficient details provided to allow replication of the method development and its use by others?

Partly

Reviewer Expertise:

NA

I confirm that I have read this submission and believe that I have an appropriate level of expertise to confirm that it is of an acceptable scientific standard.

# Reviewer report: Reviewer response for version 2

**DOI:** 10.21956/mep.21099.r33746

**Published:** 2023-06-28

**Authors:** Leila Niemi-Murola

**Affiliations:** 1 Department of Anaesthesia and Intensive Care, University of Helsinki, Helsinki, Finland

Thank you fot this revised version. I can see that the authors have revised the mansucript as suggested. I have no further comments.

Is the rationale for developing the new method (or application) clearly explained?

Partly

Is the description of the method technically sound?

Partly

Are the conclusions about the method and its performance adequately supported by the findings presented in the article?

Partly

If any results are presented, are all the source data underlying the results available to ensure full reproducibility?

Partly

Are sufficient details provided to allow replication of the method development and its use by others?

Partly

Reviewer Expertise:

Competency-based medical education

I confirm that I have read this submission and believe that I have an appropriate level of expertise to confirm that it is of an acceptable scientific standard.

# Reviewer report: Reviewer response for version 2

**DOI:** 10.21956/mep.21099.r33745

**Published:** 2023-06-20

**Authors:** Anna Chur-Hansen

**Affiliations:** 1 School of Psychology, Faculty of Health and Medical Sciences, University of Adelaide, Adelaide, SA, Australia

I confirm that my feedback - that the analysis of the qualitative data has not been conducted in a rigorous manner, stands.

Is the rationale for developing the new method (or application) clearly explained?

Partly

Is the description of the method technically sound?

Partly

Are the conclusions about the method and its performance adequately supported by the findings presented in the article?

Partly

If any results are presented, are all the source data underlying the results available to ensure full reproducibility?

Partly

Are sufficient details provided to allow replication of the method development and its use by others?

Partly

Reviewer Expertise:

Health psychology, medical education, health professional education, qualitative research

I confirm that I have read this submission and believe that I have an appropriate level of expertise to confirm that it is of an acceptable scientific standard, however I have significant reservations, as outlined above.

# Response

**Published:** 2023-06-29

**Authors:** Benjamin Moy

**Affiliations:** University of Michigan, USA

Thank you for your comments. We critically reflected on your suggestions and agree that our paper would be stronger and convey a more concise conclusion if we focused on the quantative results only. We have decided to remove the qualitative section of the manuscript (Table 3 and corresponding texts) given concerns regarding the methodology.

# Reviewer report: Reviewer response for version 1

**DOI:** 10.21956/mep.20577.r33102

**Published:** 2023-04-25

**Authors:** Anna Chur-Hansen

**Affiliations:** 1 School of Psychology, Faculty of Health and Medical Sciences, University of Adelaide, Adelaide, SA, Australia

Thank you for the opportunity to read your manuscript, which is very interesting. I have some queries and suggestions, which I have listed in the order they appear in the paper.

- In the Abstract, and in the Introduction and Discussion, I think it would be reasonable to flag that patients with limited language proficiency struggle, not only in the United States – so this is a global issue.
- Results section of Abstract – “demonstrated in the participants ability” should read “demonstrated in the participants’ ability”
- Conclusions section of the Abstract – and earlier in the Methods – and throughout the paper – I found it confusing, to understand whether these were native speakers of Mandarin, or otherwise, and it’s not until much later in the paper it becomes clear.  It would be good to make this clearer, earlier.
- For non-US readers, it is not clear (to me at least) – do students typically take languages as electives in Medical School?  Or is this unusual? 
- In the Discussion you say that this was a student led and student driven initiative, but that is not apparent from the Introduction, where it seems to be a Faculty designed and implemented intervention?
- Could the materials you have prepared for the module be made publicly available, so that others can use them?  It could be a great way to have an international collaboration going, if readers contacted you and you could pool data and design studies where you test the patients’ experiences as well as the students’?
- In the Methods – “data was” = “data were” (data is plural).
- The issue of bias, in the Methods – does it really matter?  You are not claiming representativeness, and could not possibly do so, so to me, the matter of bias is not important and could be deleted.
- For the analysis – How were the open-ended questions analysed? I think qualitative content analysis or content analysis would have been appropriate – but it seems you might have “cherry picked” quotes from the data, which is not really considered best practice. You might want to re-analyse the written responses?
- You indicate in the Results that 59 students participated – I am guessing there is no way to know how many Mandarin speaking/studying students there were at the time of the study, to give the reader a feel for response rate/participation rates?
- Apart from the clarification needed, overall an interesting piece of work – and as noted, if you could share the module for the intervention, that would be really helpful, I am sure, to others, and could result in collaborations for you and them.

Is the rationale for developing the new method (or application) clearly explained?

Partly

Is the description of the method technically sound?

Partly

Are the conclusions about the method and its performance adequately supported by the findings presented in the article?

Partly

If any results are presented, are all the source data underlying the results available to ensure full reproducibility?

Partly

Are sufficient details provided to allow replication of the method development and its use by others?

Partly

Reviewer Expertise:

Health psychology, medical education, health professional education, qualitative research

I confirm that I have read this submission and believe that I have an appropriate level of expertise to confirm that it is of an acceptable scientific standard, however I have significant reservations, as outlined above.

# Response

**Published:** 2023-06-09

**Authors:** Benjamin Moy

**Affiliations:** University of Michigan, USA

Thank you for the opportunity to read your manuscript, which is very interesting. I have some queries and suggestions, which I have listed in the order they appear in the paper. RESPONSE: Thank you for your thoughtful comments

- In the Abstract, and in the Introduction and Discussion, I think it would be reasonable to flag that patients with limited language proficiency struggle, not only in the United States – so this is a global issue.
  RESPONSE: We have reflected this comment in the introduction. Thank you for your recommendation to broaden the scope of struggles with English proficiency.
- Results section of Abstract – “demonstrated in the participants ability” should read “demonstrated in the participants’ ability”
  RESPONSE: Corrected in the text
- Conclusions section of the Abstract – and earlier in the Methods – and throughout the paper – I found it confusing, to understand whether these were native speakers of Mandarin, or otherwise, and it’s not until much later in the paper it becomes clear.  It would be good to make this clearer, earlier.
  RESPONSE: We have clarified this point in our abstract by providing context about the percentage of participants that are primary English speakers
- For non-US readers, it is not clear (to me at least) – do students typically take languages as electives in Medical School?  Or is this unusual? 
  RESPONSE: In the Discussion section, we have added context about medical school language electives.
- In the Discussion you say that this was a student led and student driven initiative, but that is not apparent from the Introduction, where it seems to be a Faculty designed and implemented intervention?
  RESPONSE: In the Discussion section, we have added context about the faculty and community partners that assisted with compilation of health information and translation of educational materials.
- Could the materials you have prepared for the module be made publicly available, so that others can use them?  It could be a great way to have an international collaboration going, if readers contacted you and you could pool data and design studies where you test the patients’ experiences as well as the students’?
  RESPONSE: The module and data sets are provided in the Dryad link located in the "Data availability" section toward the end of the paper.
- In the Methods – “data was” = “data were” (data is plural).
  RESPONSE: Corrected in the text
- The issue of bias, in the Methods – does it really matter?  You are not claiming representativeness, and could not possibly do so, so to me, the matter of bias is not important and could be deleted.
  RESPONSE: Corrected in the text
- For the analysis – How were the open-ended questions analysed? I think qualitative content analysis or content analysis would have been appropriate – but it seems you might have “cherry picked” quotes from the data, which is not really considered best practice. You might want to re-analyse the written responses?
  RESPONSE: We address that the open-ended questions were analyzed for prevailing themes that characterized the 107 unique responses. 
- You indicate in the Results that 59 students participated – I am guessing there is no way to know how many Mandarin speaking/studying students there were at the time of the study, to give the reader a feel for response rate/participation rates?
  RESPONSE: All students had completed a medical Mandarin elective and had some degree of Mandarin proficiency prior to completing the study.
- Apart from the clarification needed, overall an interesting piece of work – and as noted, if you could share the module for the intervention, that would be really helpful, I am sure, to others, and could result in collaborations for you and them. RESPONSE: The module and data sets are provided in the Dryad link located in the "Data availability" section toward the end of the paper.

# Reviewer report: Reviewer response for version 1

**DOI:** 10.21956/mep.20577.r33008

**Published:** 2023-03-20

**Authors:** Leila Niemi-Murola

**Affiliations:** 1 Department of Anaesthesia and Intensive Care, University of Helsinki, Helsinki, Finland

Thank you for the opportunity to review this interesting article focusing on virtual education of COVID-19 vaccines. In many countries, the authorities have observed low participation in the vaccinations among immigrants, and language barrier has been suspected as one of the root causes behind this phenomenon. The authors have composed an educational guide for Mandarin-speaking learners introducing key terminology and addressing commonly asked questions. The participants were medical students from seven academic medical centers across the country US.

 Methods: there is no paragraph about the questionnaire. The pre-post survey is presented in Table 2, but the free-text questions or the MCQ’s have not been described.

 Study population and Data collection. Students having completed Mandarin Chinese course were enrolled as the study population. Was the module originally constructed in English and the translated in Chinese or vice versa? What about the authors and their proficiency in Mandarin Chinese? Were the free text answers in Chinese or in English?

 Results, Figure 2. This is an interesting and illustrative figure. It would have been interesting to see all questions.

 The project contains extended data, but there is no link provided.

 I believe that this articles encourages physicians planning educational innovations for immigrants.

Is the rationale for developing the new method (or application) clearly explained?

Partly

Is the description of the method technically sound?

Partly

Are the conclusions about the method and its performance adequately supported by the findings presented in the article?

Partly

If any results are presented, are all the source data underlying the results available to ensure full reproducibility?

Partly

Are sufficient details provided to allow replication of the method development and its use by others?

Partly

Reviewer Expertise:

Competency-based medical education

I confirm that I have read this submission and believe that I have an appropriate level of expertise to confirm that it is of an acceptable scientific standard, however I have significant reservations, as outlined above.

# Response

**Published:** 2023-06-09

**Authors:** Benjamin Moy

**Affiliations:** University of Michigan, USA

Thank you for the opportunity to review this interesting article focusing on virtual education of COVID-19 vaccines. In many countries, the authorities have observed low participation in the vaccinations among immigrants, and language barrier has been suspected as one of the root causes behind this phenomenon. The authors have composed an educational guide for Mandarin-speaking learners introducing key terminology and addressing commonly asked questions. The participants were medical students from seven academic medical centers across the country US. RESPONSE: Thank you for your thoughtful comments

 Methods: there is no paragraph about the questionnaire. The pre-post survey is presented in Table 2, but the free-text questions or the MCQ’s have not been described.

 Study population and Data collection. Students having completed Mandarin Chinese course were enrolled as the study population. Was the module originally constructed in English and the translated in Chinese or vice versa? What about the authors and their proficiency in Mandarin Chinese? Were the free text answers in Chinese or in English? RESPONSE: We have added an additional paragraph in the Methods section to address these important questions. Thank you for the recommendation to provide greater context.

 Results, Figure 2. This is an interesting and illustrative figure. It would have been interesting to see all questions.

 The project contains extended data, but there is no link provided.

 RESPONSE: The data set, including all questions and responses, are provided in the Dryad link located under "Data Availability."

 I believe that this articles encourages physicians planning educational innovations for immigrants.
